# Clinical features with anti fibroblast growth factor receptor 3 (FGFR3) antibody-related polyneuropathy: a retrospective study

**DOI:** 10.1186/s12883-021-02090-2

**Published:** 2021-02-15

**Authors:** Elanagan Nagarajan, Seung Ah Kang, Carmen Holmes, Raghav Govindarajan

**Affiliations:** Department of Neurology, University of Missouri School of Medicine, Columbia, MO 65212 USA

**Keywords:** Autoimmune neuropathies, Anti-FGFR3 antibody, Sensory neuronopathies, Neuropathy

## Abstract

**Background:**

Despite its initial association with sensory neuropathies, anti-fibroblast growth factor receptor 3 (FGFR3) antibodies have been since reported with a broad range of neuropathies and clinical features. The aim of the study is to report the clinical and electro diagnostic findings in a cohort of patients with sensory or sensorimotor polyneuropathy and anti-FGFR3 antibodies.

**Methods:**

We performed a retrospective chart review to assess the clinical characteristics of patients with sensory or sensorimotor neuropathy related to FGFR3 antibodies. Descriptive statistics were reported using frequencies and percentages for categorical variables and median and interquartile range (IQR) for continuous variables.

**Results:**

This study included 14 patients (9 women) with a median age of 51.9 years (IQR 48–57). The most common presenting symptoms were painful paresthesia (100%), gait instability (42.9%), constitutional symptoms (42.9%), and autonomic symptoms (28.6%). Onset of symptoms was chronic (≥12 weeks) in eight patients (57.1%). Examination showed a distal loss of sensation to pin prick (100%), as well as impaired vibration sensation (78.6%) and proprioception (35.7%), in the distal extremities. We also observed mild weakness in the distal lower-extremities (42.9%). Three patients (21.4%) had trigeminal neuralgia, three patients (21.4%) had co-existing autoimmune disease, and one patient (7.1%) had a history of renal cell carcinoma. The mean titer of FGFR3 antibody was 14,285.71 (IQR 5000–16,750). All 14 patients produced normal results in the neuropathy workup. Nerve conduction study and electromyography showed sensory axonal neuropathy in four patients (28.6%), sensorimotor axonal neuropathy in seven patients (50%), and a normal result in three patients (21.4%). For those with a normal NCS/EMG, a skin biopsy showed a non-length-dependent small fiber neuropathy.

**Conclusions:**

Neuropathy related to FGFR3 antibodies can potentially involve small and large fibers, sensory and motor fibers, and even the trigeminal nerve, which contributes to a highly variable clinical presentation.

**Supplementary Information:**

The online version contains supplementary material available at 10.1186/s12883-021-02090-2.

## Background

Sensory neuronopathies (SNN) or ganglionopathies are a subgroup of peripheral nervous system diseases associated with the dysfunction of dorsal root ganglia (DRG) sensory neurons [1]. Like other types of sensory neuropathies, patient presentation of SNN depends on the type of nerve fiber that is affected, such as small or large fibers [2]. For instance, SNN results from damage to large afferent fibers and presents as sensory ataxia, but patients may also experience pain and paresthesia if there is damage to the small and medium-size neurons [3].

SNN is increasingly associated with an autoimmune pathogenesis, with common etiologies including paraneoplastic syndrome and Sjogren’s syndrome [1]. Though electrophysiological studies, neuroimaging, and clinical features are typically considered when making the diagnosis of SNN, these current methods fail to distinguish etiologies from one another [4, 5]. Considering that the etiology of SNN is deemed idiopathic in nearly half of patients, these current methods also fall short in capturing the complex range of etiologies [3]. Interestingly, some patients with idiopathic SNN may later develop patterns consistent with dysimmune SSN, which may suggest an underlying autoimmune mechanism [5].

Fibroblast growth factors (FGFs) are peptides that play a physiological role in the development and regeneration in the peripheral nervous system [6]. FGFs bind to their receptors, called fibroblast growth factor receptors (FGFRs), that are present in both the peripheral and central nervous system in human adults [7]. One of the receptors, FGFR3, is known to be involved in nerve regeneration and axonal development [6]. The intracellular domain of FGFR3 was found to be a target of IgG antibodies in a subgroup of patients with sensory neuropathy, particularly in idiopathic or autoimmune-related cases [7]. Still, the exact role of FGFR3 antibodies in peripheral system diseases such as neuropathies remains unclear [8].

Though anti-FGFR3 antibodies were originally associated with purely sensory-predominant neuropathies or neuronopathies, recent studies suggest that the associated neuropathy and clinical manifestation can be rather diverse and imprecise [7–10]. The aim of the study is to report the clinical and electro diagnostic findings in a cohort of patients with sensory or sensorimotor polyneuropathy and anti-FGFR3 antibodies.

## Methods

### Study design

This is a retrospective chart review of patients who presented at the University of Missouri with polyneuropathy and positive FGFR3 antibody from July 2015 to December 2017. In this timeframe, the University clinic evaluated a total of 1500 patients with sensory or sensorimotor polyneuropathy. We included length-dependent and non-length-dependent neuropathies, as well as small and large fiber neuropathies.

Testing for FGFR3 antibody in a patient was prompted if both criteria 1 and 2 were met: (1) one or more clinical features of acute to subacute onset, fluctuating course of neuropathic symptoms, asymmetric non-length dependent pattern with or without sensory ataxia, or severe neuropathic pain (even if chronic and length-dependent), and (2) the following investigations for other etiologies of neuropathy were negative: HbA1c; 2-h glucose tolerance test if HbA1c was normal; vitamin B12 with methyl malonic acid; complete metabolic panel; complete blood count; Vitamin B1 and Vitamin B6; serum protein electrophoresis with lambda kappa ratio; thyroid hormone abnormalities; paraneoplastic panel; anti-nuclear antibodies with anti-Sjogren’s-syndrome-related antigen A and B antibodies; human immunodeficiency virus (HIV) screening; hepatitis B surface antigen; and hepatitis C antibody. Furthermore, patients with either constitutional symptoms or non-length-dependent features completed a computerized tomography (CT) scan of the chest, abdomen, and pelvis with and without contrast, as well as a lumbar puncture to examine the cerebral spinal fluid (CSF). CSF studies included cell count, protein, glucose, IgG index, oligoclonal bands, flow cytometry, and cytology. Therefore, if the workup to identify other etiologies of neuropathy did not reveal a clue to the cause of the patients’ neuropathy, an autoimmune etiology was suspected and FGFR3 antibodies were subsequently tested.

Of the 1500 patients, 100 patients were tested for FGFR3 antibodies. Patients were included for review if they had sensory or sensorimotor polyneuropathy (including length-dependent, non-length-dependent, small fiber, or large fiber neuropathies), tested positive for FGFR3 antibody (antibody titer ≥3000) after being prompted by suspicion for an autoimmune etiology based on clinical features, had no other identifiable cause of neuropathy that was found during this time frame, and completed at least a 1-year follow-up. Serum samples were sent to the Neuromuscular Clinical Laboratory at Washington University School of Medicine (St. Louis, MO, USA) to test for FGFR3 antibodies according to previously described methods [11]. FGFR3 antibodies were tested by Clear Immulon Flat-Bottom Nonsterile Plates (Thermo Scientific 3455). The sera were initially tested at 1:5000 for FGFR-3. Antibody titers were calculated as the equivalent of the inverse of dilutions from ELISA values within a linear range of optical densities. Antibody-positive sera were defined as those with titers 3 standard deviations above the mean of measurements from a series of control sera tested in the Washington University Clinical Laboratory. Antibody-negative sera had titers below this level (antibody titer of 3000), or no detectable binding. The University of Missouri Institutional Review Board approved this study, which was conducted according to the University’s guidelines for retrospective studies. Informed consent was waived by the Institutional Review Board.

We retrospectively collected data on patient demographics, history of autoimmune disorders, malignancy, duration of symptoms prior to the diagnosis, and clinical symptoms from the initial evaluation. Clinical symptoms were categorized as sensory, motor, autonomic, constitutional, and/or bulbar. Nerve conduction studies (NCS) were performed in all patients in this study. NCS included motor (median, ulnar, tibial, and peroneal nerves), sensory (median, ulnar, superficial fibular, and sural nerves), and needle electromyography (EMG) as per the standard guidelines proposed by the American Association of Neuromuscular & Electro Diagnostic Medicine [12].

### Statistical analysis

Descriptive statistics were reported using frequencies and percentages for categorical variables and median and interquartile range (IQR) for continuous variables. All statistical analyses were conducted using the Statistical Package for the Social Sciences (IBM SPSS Statistic Version 21, IMB Inc., Chicago, IL).

## Results

Twenty-five patients were reported to have positive FGFR3 antibody (antibody titer ≥3000). Of these, seven patients were lost to follow-up after two visits (1–3 month follow-up) and four patients were determined to have neuropathy that was not related to FGFR3 antibody (two patients had nerve biopsy proven vasculitis; one patient had TTR amyloidosis; one patient was thought to have neuropathy due to chemotherapy). Therefore, 11 patients were excluded from the analysis criteria. In the final analysis of this study, we included a total of 14 patients who had sensory or sensorimotor polyneuropathy and positive FGFR3 antibody with a one-year follow-up.

Demographic and clinical characteristics of patients are summarized in Table 1. This sample was made up of nine women and five men, and the median age was 51.9 years (IQR 48–57). We found that painful paresthesia (100%) was the leading presenting symptom, followed by gait instability (42.9%) and constitutional symptoms (42.9%), such as weight loss and fatigue. Some patients reported autonomic symptoms (28.6%), including orthostatic hypotension and neurogenic bladder. The onset of symptoms was chronic (≥12 weeks) in eight patients (57.1%) and the median duration of symptoms prior to diagnosis was 1.95 (IQR 0.75–2.0) years.

**Table 1:** Demographic and clinical characteristics of patients with neuropathy and FGFR3 antibodies

Demographics	Age (years)	51.9 years (IQR 48–57)
	Female gender	9 (64.3%)
	Duration of symptoms (years)	1.95 (IQR 0.75–2.0)
Onset	Acute (≤ 4 weeks)	3 (21.4%)
	Subacute (4–12 weeks)	3 (21.4%)
	Chronic (≥12 weeks)	8 (57.1%)
Initial clinical manifestations	Painful paresthesia	14 (100%)
	Gait instability	6 (42.9%)
	Autonomic symptoms	4 (28.6%)
	Constitutional symptoms (weight loss, fatigue)	6 (42.9%)
Autonomic symptoms	Orthostatic hypotension	2 (14.3%)
	Neurogenic bladder	2 (14.3%)
Sensory exam findings	Distal sensory loss to pin prick in hands only	3 (21.4%)
	Distal sensory loss to pin prick in feet only	8 (57.1%)
	Distal sensory loss to pin prick in hands and feet	3 (21.4%)
	Impaired vibration sensation in distal extremities	11 (78.6%)
	Impaired proprioception in distal extremities	5 (35.7%)
Reflexes	Reduced or absent lower limb DTRs	8 (57.1%)
	Reduced or absent upper limb DTRs	2 (14.3%)
	Reduced or absent upper and lower limb DTRs	1 (7.1%)
Motor exam findings	Distal upper-extremity weakness (mild)	1 (7.1%)
	Distal lower-extremity weakness (mild)	6 (42.9%)
	No motor weakness	7 (50.0%)
Co-existing conditions	Trigeminal neuralgia	3 (21.4%)
	Diabetes mellitus type 1	1 (7.1%)
	Grave’s disease	1 (7.1%)
	Rheumatoid arthritis	1 (7.1%)
	Renal cell carcinoma	1 (7.1%)

Data are reported as n (%) and mean (interquartile range). *DTRs* deep tendon reflexes

Sensory examination showed a distal loss of sensation to pin prick in all 14 patients in this study; patients had loss of sensation to pin prick in the feet only (57%), hands only (21.4%), or both hands and feet (21.4%). We also noted impaired vibration sensation (78.6%) and proprioception (35.7%) in the distal extremities. The deep tendon reflexes (DTRs) were reduced or absent in the lower limbs in eight patients (57.1%) and in the upper limbs in two patients (14.3%). On motor examination, six patients showed mild weakness in the distal lower-extremity (42.9%) and one patient had mild weakness in the distal upper-extremity (7.1%). Seven patients (50%) were noted to have a normal motor examination.

Three patients (21.4%) also had co-existing facial pain symptoms that met the diagnostic criteria for trigeminal neuralgia, as proposed by the 3rd International Classification of Headache Disorders (ICHD-3). We saw co-existing autoimmune conditions in three patients (21.4%), including type 1 diabetes mellitus that was diagnosed in childhood, Grave’s disease, and rheumatoid arthritis. The autoimmune conditions seen in the patients of this study were controlled with medication. The patient with diabetes mellitus had an HbA1C of 6.7%. One patient (7.1%) had a history of renal cell carcinoma.

Table 2 highlights the laboratory results and NCS/EMG findings. The average titer of FGFR3 antibody was 14,285.71 (IQR 5000–16,750). All patients in this study underwent laboratory investigations as part of the neuropathy workup. Workup included HbA1c or 2-h glucose tolerance test, serum protein electrophoresis, vitamin B12, B1, and B6, thyroid function test, anti-nuclear antibodies panel, hepatitis panel, and HIV screening, which were all found to be normal. Additionally, 6 patients completed a CT scan of the chest, abdomen, and pelvis, as well as CSF analysis, which all produced normal results. The average CSF white blood cell count was 0.67 (IQR 0–1), protein was 40.3 (IQR 38.5–41.5), and glucose was 60 (IQR 58.5–61.5).

**Table 2:** Laboratory findings, CSF analysis, and NCS/EMG results

Laboratory findings	FGFR3 antibody (≥3000)	14,285.71 (IQR 5000–16,750)
CSF analysis (*n* = 6)	WBC count	0.67 (IQR 0–1)
	Protein	40.3 (IQR 38.5–41.5)
	Glucose	60 (IQR 58.5–61.5)
NCS/EMG findings	Sensory axonal neuropathy	4 (28.6%)
	Sensorimotor axonal neuropathy	7 (50%)
	Normal result	3 (21.4%)
Type of neuropathy (using results of NCS/EMG and skin biopsy)	Non-length-dependent	6 (42.9%)
	Length-dependent	8 (57.1%)

Data are reported as n (%) and mean (interquartile range). *CSF* cerebrospinal fluid, *EMG* electromyography, *FGFR3* fibroblast growth factor receptor 3, *NCS* nerve conduction studies, *WBC* white blood cell

All 14 patients underwent nerve conduction study (NCS) and electromyography (EMG), which showed abnormal results in 11 patients. Specifically, there was evidence of sensorimotor axonal neuropathy in seven patients (50%) and sensory axonal neuropathy in four patients (28.6%). Based on clinical and electrodiagnostic findings for these 11 patients, eight patients had length-dependent neuropathy and three patients had non-length-dependent neuropathy. The NCS/EMG was normal in three patients (21.4%), in which case a skin biopsy was subsequently performed (Table 3). The intraepidermal nerve fiber density in the thigh was 4.1, 3.8. and 4.2 fibers per millimeter for the three patients (mean ± SD was 21.1 ± 10.4 fibers per millimeter), which was below the 5th percentile of 5.2 fibers per millimeter. In the distal part of the leg, the intraepidermal nerve fiber density was 15.2, 14.8, and 18.2 fibers per millimeter (mean ± SD was 13.8 ± 6.7 fibers per millimeter), which was within normal limits.

**Table 3:** Skin biopsy results for three patients

Patient	Intraepidermal nerve fiber density: thigh (fibers per millimeter)	Intraepidermal nerve fiber density: distal part of leg (fibers per millimeter)
1	4.1	15.2
2	3.8	14.8
3	4.2	18.2

Mean ± SD was 21.1 ± 10.4 fibers per millimeter for the thigh (5th percentile, 5.2 fibers per millimeter). Mean ± SD was 13.8 ± 6.7 fibers per millimeter for the distal part of the leg (5th percentile, 3.8 fibers per millimeter)

## Discussion

Past studies on FGFR3-related neuropathy suggested an association between sensory neuropathy and FGFR3 antibodies, though a causal relationship is unknown [7, 9, 11]. However, the scope of these studies was restricted to measuring antibody titers in patients with pure sensory neuropathies or small fiber peripheral neuropathies, which excluded testing FGFR3 antibodies in patients with sensorimotor or motor neuropathies. Recent studies, which have expanded their inclusion criteria, shed light onto the highly variable clinical manifestation and pattern of neuropathy that is associated with FGFR3 antibodies [8, 10].

In our study, we reported the clinical features and electro diagnostic findings in patients with sensory or sensorimotor polyneuropathy related to FGFR3 antibodies. Among the 14 patients with positive FGFR3 antibodies and neuropathy included in this study, 100% of patients presented with painful paresthesia and 42.9% showed gait instability. This is higher than in previously reported data, in which painful paresthesia was reported in 13 patients (48.1%) and unsteady gait in seven patients (26%) [10]. Similarly, another study reported neuropathic pain in five patients (71.4%) and mild ataxia in only one patient (14.3%) [8]. In our study, there was mild impairment in the distal lower extremities in 42.9% of patients, which is similar to previously reported data that described 11 patients (40.1%) with either mild or severe distal lower-extremity weakness [10]. Four patients (28.6%) showed autonomic involvement in our study; other studies have reported that autonomic symptoms were present in zero patients [10] and two patients (28.6%) [8]. Though we observed trigeminal neuralgia in three patients (21.4%), this is not prevalent in recent data. However, the initial study that associated FGFR3 antibodies with sensory neuropathy reported frequent trigeminal nerve involvement [7]. Past studies did not report on constitutional symptoms, though it was present in six patients in our study. We also found co-existing autoimmune conditions in three patients (21.4%), which was similar to past reports of other autoimmune disease in six patients (22%) [10] and one patient (14.3%) [8].

EMG/NCS indicated sensory axonal neuropathy in 28.6% of patients and sensorimotor axonal neuropathy in 50% of patients in our study. Three patients (21.4%) had a normal result on the EMG/NCS, though a subsequent skin biopsy suggested a non-length-dependent small fiber neuropathy. Overall, we saw both length-dependent (57.1%) and non-length-dependent (42.9%) patterns of neuropathy but did not have any evidence of demyelination in our patients. Compared to one recent study, our study saw less variability in patterns of neuropathy as indicated by EMG/NCS. Specifically, Kovorru et al. observed sensorimotor neuropathy with purely axonal features (15%), purely demyelinating features (11%), and mixed axonal and demyelinating features (40.7%) [10]. In their study, pure sensory neuropathy was seen in 11% of patients [10]. Samara et al. reported mild/moderate sensorimotor axonal neuropathy in three patients (42.9%), demyelinating neuropathy in one patient (14.3%), and normal large fiber function in three patients (42.9%) [8].

We acknowledge that there are important limitations to our study. First, the antibody testing for FGFR3 was performed at an outside laboratory (Washington University in St. Louis). Second, the sensitivity and specificity of FGFR3 antibody testing from the laboratory has not been clearly established. Serum-specific background noise (SSBN) normalization has been shown to improve the specificity and sensitivity of the test for FGFR3 antibodies [13]. The laboratory at Washington University did not use SSBN normalization. Consequently, the increased risk of false positives, as well as using a wider inclusion criterion compared to similar published studies, may have contributed to a relatively high positivity rate of 25% (25 out of 100 patients tested positive for FGFR3 antibody). Third, we recognize that due to differences in inclusion criteria and antibody testing methodology, we are limited in the conclusions we can draw when comparing our results with other studies.

Additionally, three patients included in this study had co-existing autoimmune conditions (type 1 diabetes mellitus, Grave’s disease, and rheumatoid arthritis) that could be potential contributing factors to their neuropathy. Still, it is worth noting that the diabetes mellitus was well-controlled (HbA1c of 6.7%), and the Grave’s disease and rheumatoid arthritis were not active when the patients developed neuropathic symptoms. For this reason, we attributed the neuropathy in these three patients to FGFR3 antibodies, rather than their autoimmune conditions. We would also like to note that there were four patients in our cohort who were positive for FGFR3 antibody but were subsequently determined to have neuropathy that was not related to the antibody. In these cases, a different etiology was suspected by the clinical judgement of a neuropathy expert. Two patients had nerve biopsy proven vasculitis, one patient had TTR amyloidosis, and one patient was thought to have neuropathy due to chemotherapy; these patients were excluded from review in this study. Lastly, this study was conducted using a small sample size at a single site, which may affect the generalizability of our results. A large population-based multicenter study design that is blinded and controlled is necessary to validate our findings.

## Conclusions

The broad spectrum of clinical features and EMG/NCS results that are seen across multiple studies, including the current study, suggest that neuropathies associated with FGFR3 antibodies have the potential to involve small and large fibers, sensory and motor fibers, and the trigeminal nerve. The neuropathy associated with FGFR3 antibodies does not seem to be limited to sensory neurons, as motor nerve fiber involvement seems to be common. Previous reports also suggest that neuropathy associated with FGFR3 antibodies may be the result of damage to the axons, myelin, or both [8, 10]. Overall, this study describes the clinical and electro diagnostic findings in a well-defined group of patients with anti-FGFR3 antibody-related polyneuropathy. Our findings observed that patients with anti-FGFR3 antibody-related polyneuropathy may present with highly variable clinical features, and our paper adds to the literature on this growing subtype of neuropathy. At this time, the significance of anti-FGFR3 antibody in patients with polyneuropathy is unclear, and more studies are needed to understand their role in the pathogenesis of neuropathy.

## Supplementary Information

**Additional file 1: Supplemental Table 1A.** Patient demographic information, initial clinical manifestations, and FGFR3 titer levels. **Supplemental Table 1B.** Patient clinical characteristics in the physical exam. **Supplemental Table 1C.** CSF analysis for 6 patients.

## Data Availability

All data generated or analyzed during this study are included in this published article (and included in the supplementary data file, Table 1A-C).

## Abbreviations

CSF: Cerebral spinal fluid
CT: Computerized tomography
DTR: Deep tendon reflexes
DRG: Dorsal root ganglia
ELISA: Enzyme-linked immunosorbent assay
EMG: Electromyography
FGF: Fibroblast growth factor
FGFR: Fibroblast growth factor receptor
HIV: Human immunodeficiency virus
IQR: Interquartile range
NCS: Nerve conduction studies
SNN: Sensory neuronopathies
