# Supplementary material for: Clinical features with anti fibroblast growth factor receptor 3 (FGFR3) antibody-related polyneuropathy: a retrospective study
Source: BMC Neurol. 2021 Feb 15;21:74. doi: 10.1186/s12883-021-02090-2 (PMC7883452; doi:10.1186/s12883-021-02090-2)
Supplement: Supplementary file 1 — Additional file 1: Supplemental Table 1A. Patient demographic information, initial clinical manifestations, and FGFR3 titer levels. Supplemental Table 1B. Patient clinical characteristics in the physical exam. Supplemental Table 1C. CSF analysis for 6 patients. [file 12883_2021_2090_MOESM1_ESM.docx]

**Supplementary data**

**Supplemental Table 1A.** Patient demographic information, initial clinical manifestations, and FGFR3 titer levels.

| Patient | Age (years) | Sex (M- male, F- female) | Onset | Neuropathic pain / painful paresthesia | Gait instability | Autonomic symptoms | FGFR3 titer |
| --- | --- | --- | --- | --- | --- | --- | --- |
| 1 | 57 | M | Subacute | Yes | Yes | No | 4000 |
| 2 | 51 | F | Subacute | Yes | Yes | Yes | 5000 |
| 3 | 33 | F | Acute | Yes | Yes | Yes | 17,000 |
| 4 | 52 | M | Chronic | Yes | No | No | 28,000 |
| 5 | 71 | F | Chronic | Yes | No | No | 14,000 |
| 6 | 68 | F | Chronic | Yes | Yes | No | 5000 |
| 7 | 45 | M | Chronic | Yes | No | Yes | 13,000 |
| 8 | 47 | F | Acute | Yes | No | Yes | 15000 |
| 9 | 58 | F | Acute | Yes | No | No | 5000 |
| 10 | 57 | F | Chronic | Yes | yes | No | 40,000 |
| 11 | 57 | M | Chronic | Yes | No | No | 5000 |
| 12 | 56 | M | Chronic | Yes | Yes | No | 11,000 |
| 13 | 18 | F | Subacute | Yes | No | No | 22,000 |
| 14 | 56 | F | Chronic | Yes | No | No | 16,000 |

**Supplemental Table 1B**. Patient clinical characteristics in the physical exam.

| Patient | Pin prick | Vibration | Proprioception | Deep tendon reflexes | Strength |
| --- | --- | --- | --- | --- | --- |
| 1 | distal loss in feet | impaired in feet | normal | absent ankle | dorsiflexion 4/5 |
| 2 | distal loss in feet | impaired in feet | normal | absent ankle | dorsiflexion 4/5 |
| 3 | distal loss in feet | impaired in feet | normal | reduced ankle | dorsiflexion 4/5, eversion 4/5 |
| 4 | distal loss in feet | impaired in feet | impaired in feet | absent ankle | normal |
| 5 | loss in hands normal in feet | impaired in hands normal in feet | impaired in hands normal in feet | reduced biceps, brachioradialis, normal LL | normal |
| 6 | distal loss in feet | impaired in feet | normal | reduced ankle | normal |
| 7 | loss in hands normal in feet | impaired in hands normal in feet | impaired in hands normal in feet | absent upper limb normal LL | normal |
| 8 | distal loss in feet | impaired in feet | impaired in feet | absent ankle | dorsiflexion 4/5, eversion 4/5 |
| 9 | distal loss in feet | impaired in feet | normal | reduced ankle and knee | dorsiflexion 4/5, eversion 4/5 |
| 10 | loss in hands normal in feet | impaired in hands normal in feet | impaired in hands and normal in feet | absent in LL, reduced in UL | intrinsic hand strength 4/5 |
| 11 | loss in hands and feet | normal | normal | normal | normal |
| 12 | loss in hands and feet | normal | normal | normal | normal |
| 13 | distal loss in feet | impaired in feet | normal | reduced ankle and knee | dorsiflexion 4/5 |
| 14 | loss in hands and feet | normal | normal | normal | normal |

| Patient | WBC count | Protein | Glucose |
| --- | --- | --- | --- |
| 1 | 1 | 40 | 58 |
| 2 | 2 | 38 | 60 |
| 3 | 1 | 42 | 62 |
| 4 | 0 | 46 | 60 |
| 5 | 0 | 36 | 56 |
| 6 | 0 | 40 | 64 |

**Supplemental Table 1C**. CSF analysis for 6 patients.
